# Ivermectin sensitivity is an ancient trait affecting all ecdysozoa but shows phylogenetic clustering among sepsid flies

**DOI:** 10.1111/eva.12152

**Published:** 2014-04-10

**Authors:** Nalini Puniamoorthy, Martin A Schäfer, Jörg Römbke, Rudolf Meier, Wolf U Blanckenhorn

**Affiliations:** 1 Institute of Evolutionary Biology and Environmental Studies, University of Zürich-IrchelZürich, Switzerland; 2 Department of Biological Sciences, National University of SingaporeSingapore, Singapore; 3 ECT Oekotoxikologie GmbHFlörsheim, Germany; 4 Department of Biology, Life Sciences Complex, Syracuse University107 College Place, Syracuse, NY, 13244, USA

**Keywords:** drug resistance, dung insects, eco-toxicological traits, environmental impact studies, exaptation, ivermectin, livestock medication, phylogeny, Sepsidae

## Abstract

Avermectins are potent and popular veterinary pharmaceuticals used globally to fight parasites of livestock and humans. By disturbing ion channel transport through the membrane, avermectins are effective against endo- and ectoparasitic round and horsehair worms (Nematoida), insects, or ticks (Arthropoda), but not against Plathelminthes, including flatworms (Trematoda) and tapeworms (Cestoda), or segmented worms (Annelida). Unfortunately, excreted avermectins have strong nontarget effects on beneficial arthropods such as the insect community decomposing livestock dung, ultimately impeding this important ecosystem function to the extent that regulators mandate standardized eco-toxicological tests of dung organisms worldwide. We show that the ancient phylogenetic pattern and qualitative mechanism of avermectin sensitivity is conserved and compatible with most recent phylogenomic hypotheses grouping the Nematoida with the Arthropoda as Ecdysozoa (molting animals). At the species level, we demonstrate phylogenetic clustering in ivermectin sensitivities of 23 species of sepsid dung flies (Diptera: Sepsidae). This clustered 500-fold quantitative variation in sensitivity may indicate recent lineage-specific responses to selection, but more likely reflects pre-existing genetic variation with pleiotropic effects on eco-toxicological responses to pollutants. Regardless, our results question the common practice in eco-toxicology of choosing single test species to infer detrimental effects on entire species communities, which should ideally assess a representative taxonomic sample.

## Introduction

Avermectins are a potent and popular class of veterinary pharmaceuticals used globally to fight parasites of livestock and even humans (Õmura 2008). By disturbing ion channel transport through the membrane, avermectins are effective against endo- and ectoparasitic round and horsehair worms (Nematoida), insects, or ticks (Arthropoda), but not against Plathelminthes, including flatworms (Trematoda) and tapeworms (Cestoda), or segmented worms (Annelida) (Õmura 2008). However, excreted avermectins also have strong nontarget effects on beneficial arthropods such as the insect community decomposing livestock dung, ultimately impeding this important ecosystem function (Wall and Strong 1987; Floate et al. 2005; Jochmann et al. 2011; Lumaret et al. 2012). Systematic disturbance of dung decomposing organisms, primarily beetles, flies and earthworms, by toxic anthropogenic veterinary pharmaceuticals excreted by livestock has produced sufficiently negative effects that regulators mandate environmental risk assessments worldwide (VICH 2004; EC 2009). Eco-toxicological laboratory tests follow strict standardized guidelines (OECD 2008, 2010) and involve few, often single test species such as, for dung dwellers, the yellow dung fly *Scathophaga stercoraria* (Diptera: Scathophagidae), the face fly *Musca autumnalis* (Diptera: Muscidae), or the dung beetle *Aphodius constans* (Coleoptera: Scarabaeidae; Römbke et al. 2009, 2010a; OECD 2008, 2010). The implicit assumption in eco-toxicology is that a single test species, typically of temperate origin, represents and typifies the sensitivities of various organisms to a particular toxic substance present in natural communities (EFSA 2010; Römbke et al. 2010b). This ignores that even closely related species may have very different natural sensitivities to toxins.

Recent studies of natural populations (e.g., McKenzie and Clarke 1988; French-Constant et al. 1993; Weill et al. 2004) or experimental evolution (e.g., Lopes et al. 2008; Vogwill et al. 2012) have shown that resistance to frequently man-made toxins can evolve and spread very fast by natural selection, mediated either by point mutations within single genes or by exploiting pre-existing genetic variation in physiological pathways with sometimes unexpected pleiotropic effects on toxin sensitivity. In the simplest scenario, organisms may be naturally resistant to particular toxins (e.g., antibiotics) simply because they already bear a particular gene or genetic mechanism that may or may not have another function, and which happens to become co-opted or exapted (Gould and Vrba 1982) by chance to affect sensitivity to a novel drug (Weill et al. 2004; Allen et al. 2010; Walsh and Duffy 2013). For example, studies of *Drosophila* not only revealed that DDT resistance involves over-expression of the Rst(2)DDT locus, but also that fruit flies bearing Rst(2)DDT alleles display pre-existing cross-resistance to two other drugs used for pest control in agriculture (e.g., Daborn et al. 2001).

Comparative phylogenetic analyses can provide valuable insights about the relative importance of recent episodes of selection versus pre-existing natural genetic variation underlying drug resistance. Recent or ongoing episodes of strong selection are expected to diminish the signal of shared common ancestry, which in the extreme will annihilate any correlation between phylogeny and the trait (Losos 1999). Moreover, when comparing the sensitivity of organisms to particular toxins, we would expect a pattern in which populations or species inhabiting the same geographic region share a higher propensity for being drug resistant, even if they are not closely related to each other, due to their common history of exposure to a particular toxin. In contrast, strong phylogenetic clustering of toxin sensitivity would rather suggest co-option of pre-existing genetic variation prior to exposure to the toxin.

We have previously assessed the sensitivity, in terms of mortality, to the prominent and widely used parasiticide ivermectin for 23 temperate (Europe, North America) and tropical (Asia, Central America) sepsid dung fly (sub)species in an ecotoxicological study (Blanckenhorn et al. 2013). All species used are ecologically bound to fresh feces of large mammals or, rarely, waterfowl, in which larval development takes place. In nature, these species can be collected from fresh cow dung, often several species on the same cowpat, which is also the breeding substrate of choice in our laboratory. Here, we map our ecotoxicological data onto the most recent sepsid phylogeny based on 10 genes (Lei et al. 2013) to explore the compatibility of the ancient phylogenetic pattern of avermectin sensitivity with most recent phylogenomic hypotheses (Burda et al. 2008; Telford and Copley 2011) grouping the Nematoida together with the Arthropoda as Ecdysozoa (molting animals). We further investigate more recent phylogenetic patterns of ivermectin susceptibility among closely related sepsid flies. Our results suggest that ivermectin sensitivity in sepsid flies evolved largely independently of exposure to avermectins.

## Methods

We worked with 23 sepsid species that were identified using Pont and Meier (2002) and SepsidNet (Ang et al. 2013). The sample included differentiated North American and European subspecies (populations) of *Sepsis neocynipsea* and *S. punctum* (Puniamoorthy et al. 2012). For 7 temperate *Sepsis* species (*Sepsis cynipsea, S. fulgens, S. neocynipsea*, *S. orthocnemis, S*. *punctum, S. thoracica,* and *S. violacea;* Table S1), we collected multiple (2–5) populations, which originally served for estimating within-species variation in sensitivity (Blanckenhorn et al. 2013); these data were averaged in our main analysis here. Depending on availability, we also collected two or more of our test species from the same sites (i.e., populations): Vienna, Austria (5 spp); Zurich, Switzerland (6 spp); Sierra Nevada, Spain (2 spp); Tartu, Estonia (2 spp); Umbria, Italy (3 spp); Calabria, Italy (2 spp). This allows comparing systematic geographic variation in ivermectin sensitivity across species, which may reflect local adaptation to past drug exposure. All flies used were originally caught by ourselves in the wild at the various sites specified in Table S1, on or around cow dung. Ideally, multiple (>10) females for each species were caught at each site of origin, which were subsequently kept in our laboratories in Zurich and/or Singapore for multiple generations using standard methods (e.g., Puniamoorthy et al. 2012), either in large population containers or in smaller containers as iso-female lines (i.e., offspring of one field-caught female).

Laboratory tests were performed in three temporal blocks (2008, 2009, and 2011) using standard toxicological methods with six ivermectin concentrations (0.21, 0.66, 2.08, 6.57, 20.75, and 65.7 *μ*g ivermectin/kg dung fresh weight) plus water and acetone controls (five replicates per concentration; complete methods and toxicological results are reported in Blanckenhorn et al. 2013; Table S1). Separately for each species or population, lethal ivermectin concentrations causing 50% larva-to-adult mortality (LC50) plus their 95% confidence intervals were estimated by regressing logit-transformed emergence proportions against log_10_(ivermectin concentration) (ToxRat 2003). For comparison, we additionally estimated adult body size (head width in mm) and egg-to-adult development time (in days) of all flies in the control (i.e., water and acetone) treatments (see Table S1), two life history traits that are generally subject to natural selection (e.g., Blanckenhorn et al. 2007), and which are nonlethally reduced and prolonged, respectively, by ivermectin (Römbke et al. 2009; Blanckenhorn et al. 2013).

**Figure 2:**
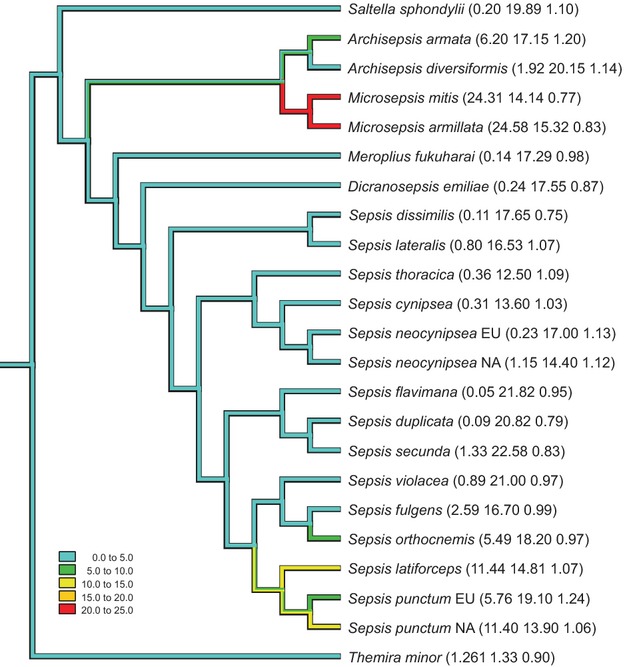
Ivermectin sensitivity (lethal concentration at which 50% of individuals die LC50, in *μ*g ivermectin/kg fresh dung), mean development time (days), and mean head width (mm) (in parentheses) for 23 sepsid dung fly (sub)species mapped onto their phylogeny (females and males combined; cf. Table S1). Tree branches are colored according to the LC50 values (see legend) to visualize the nonrandom sensitivity pattern.

We used *Mesquite* (Maddison and Maddison 2011) to map mean ivermectin LC50, body size, and development time data as continuous traits onto the most recent sepsid phylogeny (Lei et al. 2013) to reveal potential phylogenetic clustering of ivermectin sensitivity at the (sub)species taxonomic level. Phylogenetic clustering of our three traits was independently tested via 1000-fold randomization of trait values without replacement across species on the given phylogeny, yielding a *P*-value indicating how extreme the actual distribution of species on the phylogeny (Fig. 2) is relative to all other possible, randomly assembled distributions. To investigate potential concerted evolution by natural selection of life history traits with ivermectin sensitivity, we further computed correlations between the three traits using independent contrasts (CAIC: Purvis and Rambaut 1995).

At the ancient phylogenetic level, we mapped avermectin sensitivity onto the most recent and now widely accepted phylogenomic hypothesis grouping the Nematoida together with the Arthropoda as Ecdysozoa (molting animals: Burda et al. 2008; Telford and Copley 2011).

## Results

**Figure 1:**
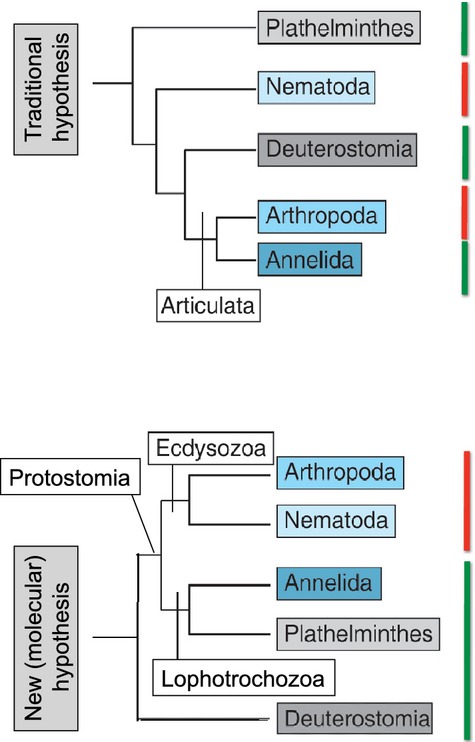
Traditional (top) and new (bottom) phylogenetic hypotheses for the ancient relationships between invertebrate taxa (adapted from Burda et al. 2008). The pattern of ivermectin sensitivity (red = sensitive and green = nonsensitive bars) more parsimoniously agrees with the most recent phylogenomic hypothesis.

The modern sister group relationship of Arthropoda and Nematoida (Ecdysozoa hypothesis) suggests a single origin of avermectin sensitivity in animals, whereas the formerly hypothesized sister group relationship of Arthropoda and Annelida (Articulata hypothesis) would require assumption of multiple origins (Fig. 1; adapted from Burda et al. 2008).

At the species level, we found up to 500-fold variation in the mean LC50 among the tested related sepsid flies (Fig. 2). The variation in sensitivity is nonrandomly distributed on the sepsid phylogeny as revealed by trait value randomization without replacement (*P* < 0.002). At least two clades, *Archisepsis* plus *Microsepsis* from Central America and the species of the *Sepsis punctum* group that are widespread in Eurasia and North America, have significantly lower ivermectin sensitivities, as indicated by higher LC50 values in Fig. 2. Body size is also nonrandomly distributed (i.e., clustered: *P* < 0.032), while development time is seemingly randomly distributed on the phylogeny (*P* < 0.154; Fig. 2). Importantly, neither life history trait correlates with ivermectin sensitivity (|*r*| < 0.3; *P* > 0.2), so their patterns evolved independently.

When comparing ivermectin sensitivity (logit-transformed LC50 value) among sites while controlling for species in a two-way anova for the subset of seven species collected from more than one site in Europe (seven sites), as well as when considering all species, no systematic geographic variation was apparent *F*_6,14_ = 1.38, *P* = 0.287 (site by species interaction removed due to nonsignificance; the same results when including all species and sites: *F*_12,14_ = 1.57, *P* = 0.208). For the subset, variance component analysis revealed approximately 4.5 times more variance among species (4.8) than was observed among sites (1.1).

## Discussion

Our study shows that eco-toxicological responses to pollutants, exemplified by the LC50 values used here, can and should be treated as traits subject to evolution that may follow phylogenetic patterns. At the ancient phylogenetic level, the pattern of avermectin sensitivity of invertebrates conforms to the modern phylogenomic hypothesis grouping the Nematoida together with the Arthropoda as Ecdysozoa (molting animals), rather than the traditional hypothesis grouping Annelida and Arthropoda as Articulata. Retrospectively, phylogenetic relationships can thus explain why avermectins unfortunately do not work against major parasitic helminth groups such as flatworms and tapeworms (Plathelminthes), but only against roundworms (Nematoida), and why they at the same time negatively affect Arthropoda, which comprise numerous nontarget beneficial species such as the beetles and flies decomposing livestock dung treated here: Only the two latter groups molt, and apparently avermectins disturb ion channel transport through the membrane especially during molting (Õmura 2008). Fortunately, avermectins also do not strongly affect annelids such as earthworms (Lumaret et al. 2012), which also play a very important role as decomposers of vertebrate dung (Holter 1979; Lumaret et al. 2012), and this is again consistent with the most recent Ecdysozoa hypothesis depicted in Fig. 1. These patterns could not have been predicted by the previously accepted Articulata hypothesis, showing that phylogenetic information is useful for predicting large-scale patterns of resistance to toxins. Eco-toxicology thus supports recent findings in phylogenomics (Telford and Copley 2011), and important new insights were gained here from analyzing resistance data in a phylogenetic context.

A highly significant phylogenetic signal in ivermectin sensitivity was also revealed in a clade of 23 dung-dwelling sepsid flies by trait randomization among species on our phylogeny. Two subclades independently show lower susceptibility to ivermectin (Fig. 2), the Neotropical *Microsepsis* and *Archisepsis* species, as well as several Eurasian *Sepsis* species of the *punctum* group (*S. violacea*, *fulgens*, *orthocnemis*, *latiforceps*, *punctum*: Pont and Meier 2002). As outlined in the Introduction, this phylogenetic pattern can be explained either by natural selection against ivermectin sensitivity, that is, by greater adaptability, or by pre-existing genetic variation that evolved in a different context sometime in the distant past and which later pleiotropically affected toxin resistance in some groups, but not others (i.e., an exaptation: Gould and Vrba 1982). Ivermectin has been in use only for about 40 years (Õmura 2008), corresponding to roughly 500 sepsid fly generations with a generation time of ca. 3 weeks. That insects with large effective populations can rapidly evolve resistance to insecticides is a well-known phenomenon from pest control (McKenzie and Clarke 1988; French-Constant et al. 1993; Weill et al. 2004; Lopes et al. 2008; Vogwill et al. 2012), and this could also be the case in sepsid flies. Indeed, a substantial fraction of the total variation in ivermectin resistance in sepsid flies can be attributed to population differentiation (Blanckenhorn et al. 2013). However, there are several reasons why this scenario seems unlikely. The less sensitive species in the *punctum* group regularly co-occur on the same pastures with several other *Sepsis* species that are very sensitive to ivermectin (e.g., *S. cynipsea* or *flavimana*; Fig. 2). Recurrent substance treatment in such regions should simultaneously select against sensitivity of all sympatric species from various lineages, not only in some. Explaining the obtained pattern by natural selection for ivermectin resistance would require the parallel fixation of mutations in several reproductively isolated species or populations within very short time, producing geographic patterns of sensitivity. Yet we found that variation between species in ivermectin sensitivity is about 4–5 times greater than variation between sites commonly harboring populations of several of these species, although we readily admit that our taxon and population sampling was opportunistic and not ideal for systematically examining geographic variation in susceptibility. Unfortunately, we have no information about ivermectin use at the various sites, as the substance is not controlled everywhere. We know only that ivermectin use varies locally, even by farmer, and that the flies' dispersal distance likely transcends and homogenizes any regional patterns of substance use. Therefore, while the nonrandom phylogenetic sensitivity pattern we found does not principally exclude recent rapid evolution of resistance to man-made toxins by natural selection, we do not think that it is the most parsimonious explanation for our data.

There are additional reasons why a phylogenetic explanation of the obtained pattern is more reasonable. It is known that organisms can be ‘naturally’ resistant to novel toxins without ever having been exposed to them (Weill et al. 2004; Allen et al. 2010; Walsh and Duffy 2013). Thus, it is probable that the nonrandom pattern of ivermectin sensitivity in sepsid dung flies evolved as a by-product correlated with some unknown trait that changed in the distant past, exemplifying a pre-adaptation or exaptation (Gould and Vrba 1982) conferring a selective advantage only *post hoc* in pastoral habitats created by humans. The connection to the ancient phylogenetic level of ivermectin sensitivity to molting (Fig. 1), an evolutionary novelty at that time, can perhaps also be interpreted as such an exaptation, in this case conferring a disadvantage *post hoc*. Some further, circumstantial but anecdotal evidence supports our evolutionary interpretation. First, the species most likely exposed to ivermectin should be the cow dung specialists *Sepsis cynipsea* and *S. neocynipsea* that are most abundant in Europe and North America, respectively, which however are two of the most sensitive species both in the laboratory (Blanckenhorn et al. 2013; Fig. 2) and in the field worldwide (Madsen et al. 1990; Floate 1998; Iwasa et al. 2005). Second, one of the lesser susceptible *Archisepsis* species (Fig. 1; Table S1) was collected in a nature reserve on an island in Panama (Barro Colorado) that has never been used agriculturally. Third, ivermectin has been isolated from the producing fungus *Streptomyces avermectinus* in Japan about 40 years ago (Õmura 2008), so the common ancestors of the two least sensitive sepsid groups in Fig. 2 could have plausibly evolved ivermectin resistance in the distant past in response to natural contact with that fungus. This is again unlikely, however, because the substance, and the fungus, so far has been found only this one time and nowhere else (Õmura 2008).

In summary, we conclude that the nonrandom pattern of ivermectin sensitivity only affecting molting animals (Ecdysozoa in Fig. 1), as well as that found for sepsid dung flies (Fig. 2), likely indicates exaptations (Gould and Vrba 1982) at two different levels: first conferring a selective disadvantage to all sensitive molting animals, and in a second step, a selective advantage to some species *post hoc* in pastoral habitats created by humans. Susceptibility to natural or man-made toxins therefore need not necessarily be adaptations, as is often assumed (e.g., Tack et al. 2012). Furthermore, at a more practical level in the realm of toxicology, the strong phylogenetic signal of ivermectin sensitivity we found implies that any particular species cannot possibly be representative when chosen for assessing toxicity of substances affecting the dung community. This makes choice of any test species in the context of environmental risk assessments of veterinary pharmaceuticals particularly delicate (Blanckenhorn et al. 2013). One obvious solution to the problem is to mandate use of several test species based on diverse phylogenetic taxon sampling, or even the dung community as a whole (Floate et al. 2005; Jochmann et al. 2011), thus extending the registration process already required for these drugs (VICH 2004). In this context, the issue of regionalization of substance control should also be considered, as is already being discussed (EFSA 2010).

## Data archiving statement

Data for this study are available in the supporting information.

## Supporting Information

**Table S1.** Proportion of flies emerged from the control treatments (water and acetone) for all 47 tests using 21 sepsid species, estimated lethal concentration at which 50% of the flies died (LC50, in μg ivermectin/kg wet dung) with 95% confidence limits, and mean ± SE development time and body size for male and female sepsids (Nm, Nf = total number of males, females) (species averages with SD in italics).

## References

[b1] Allen HK, Donato J, Wang HH, Cloud-Hansen KA, Davies J, Handelsman J (2010). Call of the wild: antibiotic resistance genes in natural environments. Nature Reviews Microbiology.

[b2] Ang Y, Puniamoorthy J, Pont AC, Bartak M, Blanckenhorn WU, Eberhard WG, Puniamoorthy N (2013). A plea for digital reference collections and other science-based digitization initiatives in taxonomy: sepsidnet as exemplar. Systematic Entomology.

[b3] Blanckenhorn WU, Dixon AFG, Fairbairn DJ, Gibert P, van der Linde K, Meier R, Nylin S (2007). Proximate causes of Rensch's rule: does sexual size dimorphism in arthropods result from sex differences in development time?. The American Naturalist.

[b4] Blanckenhorn WU, Puniamoorthy N, Schäfer MA, Scheffczyk A, Römbke J (2013). Standardized laboratory tests with 21 species of temperate and tropical sepsid flies confirm their suitability as bioassays of pharmaceutical residues (ivermectin) in cattle dung. Ecotoxicology and Environmental Safety.

[b5] Burda H, Hilken G, Zrzavy J (2008). Systematische Zoologie.

[b6] Daborn P, Boundy S, Yen J, Pittendrigh B, French-Constant R (2001). DDT resistance in *Drosophila* correlates with Cyp6 g1 over-expression and confers cross.resistance to the neonicotinoid imidacloprid. Molecular Genetics and Genomics.

[b7] European Community (EC) (2009). Commission Directive 2009/9/EC Amending Directive 2001/82/EC of the European Parliament and of the Council on the Community Code Relating to Medicinal Products for Veterinary use.

[b8] European Food Safety Authority (EFSA) (2010). Scientific Opinion on the development of a Soil Ecoregions concept. The EFSA Journal.

[b9] Floate KD (1998). Off-target effects of ivermectin on insects and on dung degradation in southern Alberta, Canada. Bulletin of Entomological Research.

[b10] Floate KD, Wardhaugh KG, Boxall ABA, Sherratt TN (2005). Faecal residues of veterinary pharmaceuticals: non-target effects in the pasture environment. Annual Review of Entomology.

[b11] French-Constant RH, Rocheleau TA, Steichen JC, Chalmers AE (1993). A point mutation in a *Drosophila* GABA receptor confers insecticide resistance. Nature.

[b12] Gould SJ, Vrba S (1982). Exaptation – a missing term in the science of form. Paleobiology.

[b13] Holter P (1979). Effect of dung-beetles (Aphodius spp) and earthworms on the disappearance of cattle dung. Oikos.

[b14] International Cooperation on Harmonisation of Technical Requirements for Registration of Veterinary Medicinal Products (VICH) (2004). Environmental Impact Assessment for Veterinary Medicinal Products – Phase II. Guidance.

[b15] Iwasa M, Maruyama M, Nakamura E, Yamashita N, Watanabe A (2005). Effects of ivermectin on target and non-target dung-breeding flies (Diptera) in cattle dung pats. Medical Entomology and Zoology.

[b16] Jochmann R, Blanckenhorn WU, Bussière L, Eirkson CE, Jensen J, Kryger U, Lahr J (2011). How to test nontarget effects of veterinary pharmaceutical residues in livestock dung in the field. Integrated Environmental Assessment and Management.

[b17] Lei Z, Ang SHA, Srivathsan A, Su KFY, Meier R (2013). Does better taxon sampling help? A new phylogenetic hypothesis for Sepsidae (Diptera: Cyclorrhapha) based on 50 new taxa and the same old mitochondrial and nuclear markers. Molecular Phylogenetics and Evolution.

[b18] Lopes PC, Sucena E, Santos ME, Magalhaes S (2008). Rapid experimental evolution of pesticide resistance in *C. elegans* entails no costs and affects the mating. PLoS ONE.

[b19] Losos JB (1999). Uncertainty in the reconstruction of ancestral character states and limitation on the use of phylogenetic comparative methods. Animal Behaviour.

[b20] Lumaret JP, Errouissi F, Floate K, Roembke J, Wardhaugh K (2012). A review on the toxicity and non-target effects of macrocyclic lactones in the terrestrial and aquatic environment. Current Pharmaceutical Biotechnology.

[b21] Maddison WP, Maddison DR (2011). http://mesquiteproject.org.

[b22] Madsen M, Overgaard-Nielsen B, Holter P, Pedersen OC, Jespersen JB, Vagn-Jensen KM, Nansen P (1990). Treating cattle with ivermectin: effects on the fauna and decomposition of dung pats. Journal of Applied Ecology.

[b23] McKenzie JA, Clarke GM (1988). Diazon resistance, fluctuating asymmetry and fitness in the Australian sheep blowfly *Lucilia cuprina*. Genetics.

[b24] Õmura S (2008). Ivermectin: 25 years and still going strong. International Journal of Antimicrobial Agents.

[b25] Organisation for Economic Co-Operation and Development (OECD) (2008). OECD Guidelines for the Testing of Chemicals. Determination of Developmental Toxicity of a Test Chemical to Dipteran Dung Flies (Scathophaga Stercoraria L. (Scathophagidae), Musca Autumnalis De Geer (Muscidae)).

[b26] Organisation for Economic Co-Operation and Development (OECD) (2010). Guidance Document on the Determination of the Toxicity of a Test Chemical to the Dung Beetle Aphodius Constans.

[b27] Pont AC, Meier R (2002). The Sepsidae (Diptera) of Europe. Fauna Entomologica Scandinavica.

[b28] Puniamoorthy N, Blanckenhorn WU, Schaefer MA (2012). Differential investment in pre- versus post-copulatory sexual selection reinforces a cross-continental reversal of sexual size dimorphism in *Sepsis punctum* (Diptera: Sepsidae). Journal of Evolutionary Biology.

[b29] Purvis A, Rambaut A (1995). Comparative-analysis by independent contrasts (CAIC) - an Apple-Macintosh application for analyzing comparative data. Computational and Applied Biosciences.

[b30] Römbke J, Floate KD, Jochmann R, Schäfer MA, Puniamoorthy N, Knäbe S, Lehmhus J (2009). Lethal and sublethal toxic effects of a test chemical (Ivermectin) on the yellow dung fly (*Scathophaga stercoraria*) based on a standardized international ring test. Environmental and Toxicological Chemistry.

[b31] Römbke J, Barrett K, Blanckenhorn WU, Hargreaves T, Kadiri N, Knäbe S, Lehmhus J (2010a). Results of an international ring test with the dung fly *Musca autumnalis* in support of a new OECD test guideline. Science of the Total Environment.

[b32] Römbke J, Jänsch S, Meier M, Hilbeck A, Teichmann H, Tappeser B (2010b). General recommendations for soil ecotoxicological tests suitable for the Environmental Risk Assessment (ERA) of Genetically Modified Plants (GMPs). Integrated Environmental Assessment and Management.

[b33] Tack AJM, Thrall PH, Barret LG, Burdon JJ, Laine AL (2012). Variation in infectivity and aggressiveness in space and time in wild host–pathogen systems: causes and consequences. Journal of Evolutionary Biology.

[b34] Telford MJ, Copley RR (2011). Improving animal phylogenies with genomic data. Trends in Genetics.

[b35] ToxRat (2003). Software for the Statistical Analysis of Biotests.

[b36] Vogwill T, Lagator M, Colegrave N, Neve P (2012). The experimental evolution of herbicide resistance in *Chlamydomonas reinhardtii* results in a positive correlation between fitness in the presence and absence of herbicides. Journal of Evolutionary Biology.

[b37] Wall R, Strong L (1987). Environmental consequences of treating cattle with the antiparasitic drug ivermectin. Nature.

[b38] Walsh F, Duffy B (2013). The culturable soil antibiotic reistome: a community of multi-drug resistant bacteria. PLoS ONE.

[b39] Weill M, Berthomieu A, Berticat C, Lutfalla G, Nègre V, Pasteur N, Philips A (2004). Insecticide resistance: a silent base prediction. Current Biology.

